# Effects of different HTO correction angles on ankle joint biomechanics in 3D varus knee model: a patient-specific simulation study

**DOI:** 10.3389/fbioe.2026.1808741

**Published:** 2026-08-25

**Authors:** Yinghui Zhu, Adeel Anwar, Mengke Ren, Yufang Zhang, Weiguo Zhang, Zhen Zhang

**Affiliations:** 1 Department of Orthopaedic Surgery, The First Affiliated Hospital of Dalian Medical University, Dalian, China; 2 Department of Orthopaedic Surgery, Dalian No. 3 People’s Hospital, Dalian, China; 3 Department of Engineering Mechanics, Dalian University of Technology, Dalian, China; 4 Department of Mechanical Engineering, Zhengzhou Railway Vocational and Technical University, Zhengzhou, Henan, China

**Keywords:** ankle joint, finite element analysis, knee osteoarthritis, mechanical axis, subtalar joint, varus knee, high tibial osteotomy

## Abstract

**Objective:**

The mechanical axis in the varus knee deteriorates normal ankle joint biomechanics. Consequently, degrees of correction angles may also alter ankle stresses. This study aimed to elaborate the stress changes in articular cartilages and pressure in the subtalar joint of the ankle joint using finite element analysis (FEA) with different osteotomy correction angles.

**Methods:**

Computed tomography and magnetic resonance images were propagated to get a full leg model that included the knee and ankle joints. Computer-aided design (CAD) software was used to simulate osteotomy. Then, correction axes were carried out. Osteotomy gaps were filled to mimic bone healing. Model A represents the varus knee. Models B, C, D, and E represent osteotomy models with a neutral axis and 3.5°, 5.5°, and 7.5° over-corrected valgus angles, respectively. Simulated loads were applied to the femoral head.

**Results:**

The mean stress in tibial cartilage (71.69 MPa) in model A was significantly reduced to 36.17 MPa, 34.55 MPa, 34.14 MPa, and 32.25 MPa in high tibial osteotomy (HTO) correction models B, C, D, and E, respectively (*p* < 0.001). Contrarily, the mean von Mises stress (VMS) of 23.81 MPa in model A was reduced to 19.74 MPa and to 18.60 MPa in models C and D, respectively (*p* < 0.05) in talar cartilage. The shear stress analysis of tibial cartilage showed that the peak shear stress of 8.289 MPa was reduced to 5.203 MPa in the neutral axis model (*p* < 0.05) and further decreased to 4.639 MPa, 4.569 MPa, and 4.666 MPa in models with a neutral axis and 3.5°, 5.5°, and 7.5° valgus (*p* < 0.05), respectively. The subtalar joint pressure was higher in over-corrected models with 5.5° and 7.5° valgus angles (*p* < 0.05), but in the 3.5° valgus model, this increase was non-significant (*p* > 0.05).

**Conclusion:**

The varus knee model and its corrective osteotomy had a definitive impact on the ipsilateral ankle joint and the hindfoot. Ankle joint stresses were significantly reduced in the 3.5° valgus model. Subtalar pressure was lateralized in varus models, whereas it was medialized in valgus correction models. Surgeons should consider these compensatory ankle joint biomechanical alterations before planning HTO.

## Introduction

Knee osteoarthritis (KOA) is one of the most common degenerative diseases across the globe (Cui et al., 2020; Cross et al., 2014). The knee is a complex and most vulnerable joint because it provides rapid movements while bearing high loads during daily activities. Biomechanically, approximately 75% of the body weight passes through the medial aspect of the knee (Andriacchi, 1994; Périé and Hobatho, 1998). Therefore, the medial knee compartment is affected in most KOA cases (Hernborg and Nilsson, 1977). A substantial change in lower extremity biomechanics may redistribute the load across the articular cartilage, and it is assumed to be an important factor in varus knee deformity (Heijink et al., 2011). In the long run, the overloaded medial knee compartment in varus deformity may exacerbate ankle joint pain (Hubbard et al., 2010). Some recent clinical studies concluded that ankle and foot pain are induced by knee deformities (Norton et al., 2015; Okamoto et al., 2016). The ankle pain and tenderness are common chief complaints in orthopedics clinics and may interfere with the normal range of motion and consequently, the quality of life (Nakamura et al., 2016; Murray et al., 2018). However, it is not completely evident how the ankle and hindfoot compensate for knee deformity (Chandler and Moskal, 2004; Desai et al., 2007; Keenan et al., 1991; Mullaji and Shetty, 2011).

It is advocated that in varus knee deformity, the hindfoot settles in a valgus position using an eversion shift (Saltzman and El-Khoury, 1995; Reilingh et al., 2010). The effect of knee deformities and treatment strategies on the ankle joint remained the point of discussion in some past studies (Krause et al., 2017; Choi et al., 2018; Oh et al., 2023; Oh et al., 2024; Kawashima and Takagi, 2022; Shim et al., 2023). However, the biomechanical effect of different corrective osteotomy models (Akizuki et al., 2008; Coventry, 1965; Dugdale et al., 1992; Flecher et al., 2006; Kettelkamp et al., 1976; Koshino et al., 2004; Papachristou et al., 2006; Tang and Henderson, 2005) on the ankle joint is lacking in the recent literature. We hypothesized that varus knee deformity would alter ankle stress distribution and subtalar pressure, and that correction to a mild valgus alignment would reduce ankle joint stress. We further hypothesized that excessive valgus overcorrection would not provide additional ankle biomechanical benefit and might increase subtalar pressure or shift stress medially. Accordingly, the aim of this finite element study was to explore the influence of varus knee and corrective osteotomy models (with different valgus correction angles) on the stress variations that occurred in the ankle joint using the patient-specific 3D models. Finally, the statistical analysis was performed to verify the significant difference between different study models.

## Materials and methods

### Three-dimensional modeling and establishment of the knee-ankle complex

**FIGURE 1:**
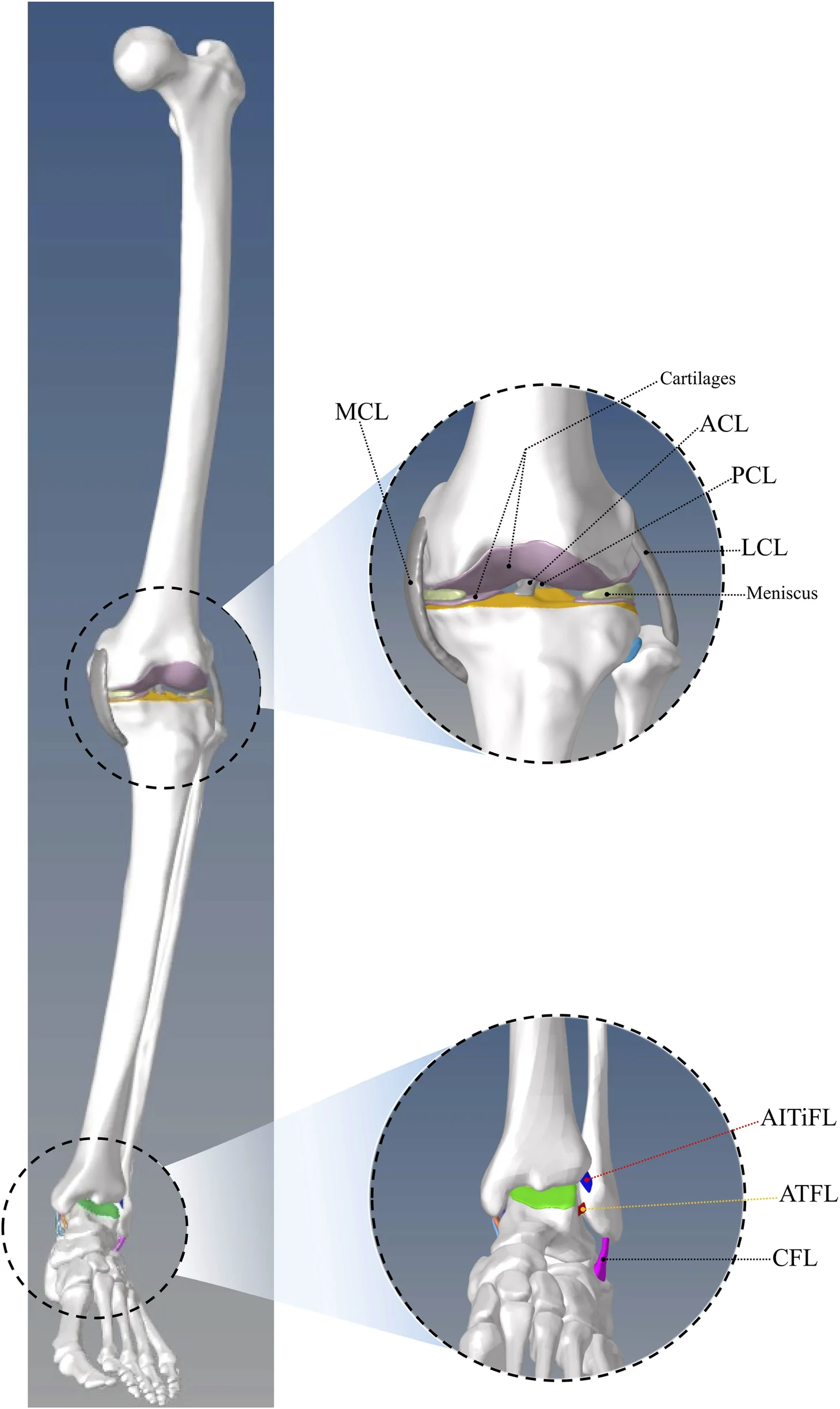
Initial 3D full leg model including the foot.

To establish three-dimensional (3D) knee and ankle models, CT scan and MRI images of the left full leg, including the foot, from a 41-year-old female of 70 kg weight with a medial compartment osteoarthritis (OA) knee were used. CT scan data in DICOM (Digital Imaging and Communications in Medicine) format was imported into Mimics 19.0 software (Materialise, Leuven, Belgium) to reconstruct the surface point cloud geometry of the left leg and foot. Then, Initial Graphics Exchange Specification (IGES) structural files were exported. These IGES files were used to transfer the initial data into the reverse engineering software program, Geomagic Studio 2015 (Raindrop Company, United States), for 3D optimization. The processing in Geomagic Studio converted the point clouds to volumetric bone structures (3D models). After that, Standard for the Exchange of Product Model Data (STEP) files were exported for SolidWorks 2012 (DS Solid Works Corp., United States) processing and modeling. The coordinate axes of the assembled model were aligned as the X-axis pointed laterally (medial to lateral femoral condyles in the knee joint and medial to lateral malleolus in the ankle joint), the Y-axis pointed posteriorly (anterior to posterior), and the Z-axis pointed upward (ankle to knee direction). The initial 3D model is shown in Figure 1.

**FIGURE 2:**
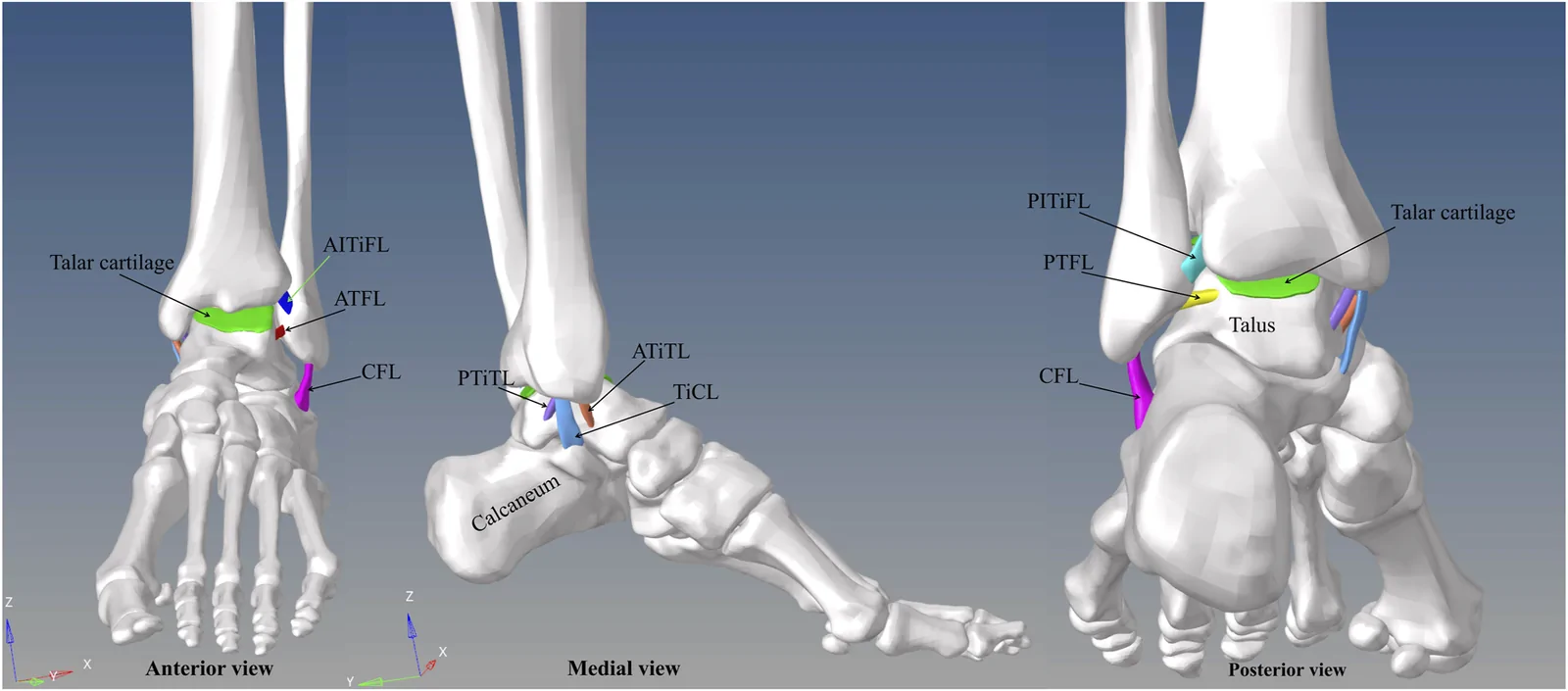
Close-up view of the ankle joint elaborating different ligaments.

The stereolithography (STL) format of these models was utilized in 3Matic software to build soft tissues of the knee and ankle joints using MRI reference points. The soft tissues of the knee included: femoral cartilage, tibial plateau cartilages (both medial and lateral), tibiofibular joint (TFJ), collateral ligaments of the knee; medial collateral ligament (MCL), lateral collateral ligament (LCL), and cruciate ligaments: anterior cruciate ligament (ACL), posterior cruciate ligament (PCL). Similarly, soft tissues of the ankle joint included cartilages of the tibial plafond and talar cartilages. The ligaments around the ankle were the tibiocalcaneal ligament (TiCL), anterior tibiotalar ligament (ATiTL), posterior tibiotalar ligament (PTiTL), anterior talofibular ligament (ATFL), posterior talofibular ligament (PTFL), anterior inferior tibiofibular ligament (AITiFL), and posterior inferior tibiofibular ligament (PITiFL), as shown in Figure 2.

**FIGURE 3:**
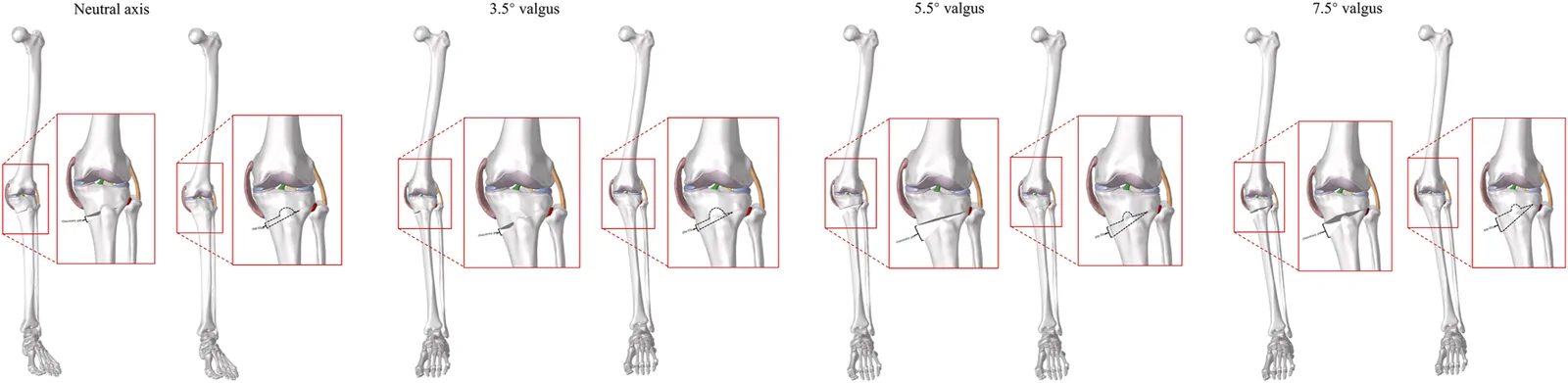
Different models showing correction angles and ankle joint position.

To simulate high tibial osteotomy, the osteotomy cuts were induced using computer-aided design (CAD) software SolidWorks in the upper tibia (Park et al., 2024; Chen et al., 2019). The lower part of the leg and ankle joint were moved to get the required correction angles of neutral axis and over-corrected angles of 3.5°, 5.5°, and 7.5° valgus. Then the osteotomy gaps were filled to mimic bone healing, as shown in Figure 3. Study models were model A, a varus knee model; B, with a normal mechanical axis; C, with a 3.5° valgus; and D and E, with 5.5° and 7.5° valgus, respectively.

### Material properties and finite element processing

These five study models were meshed using HyperMesh (HyperWorks, 19.0, Altair Engineering, Inc, United States). The cortical and cancellous parts of the tibiae were separated in HyperMesh during the meshing process. A 2D auto-meshing shell was initially utilized for cortical bones, then tetrahedral C3D10 elements (3D tetra-meshing) were established to mesh the cancellous portion of bones. In this process, a quadratic tetrahedral mesh was established in the cortical and cancellous parts. The mesh sizes of 1 mm and 0.5 mm were selected for bones and soft tissues (joint cartilages and ligaments) after mesh convergence, respectively (Supplementary Figures S1A,B). INP files of 3D models were imported into finite element analysis (FEA) software Abaqus (Abaqus 6.14, Simulia Corp., United States). In Abaqus, different parts of the models were assembled into a full leg model, and interactions were conducted between these parts of the full leg model, including the ankle joint.

The components of the knee joint, that is, the bony components: femur, tibia, and fibula, and soft tissue components: cartilages, menisci, and ligaments (ACL, PCL, MCL, and LCL), were simulated according to the material properties documented in the previous literature as given in Table 1. Bones were assumed to behave as homogeneous, isotropic, and linearly elastic material (Anwar et al., 2020; Li et al., 2024). The interactions between the cartilage and bones were fully bonded tie interactions (Anwar et al., 2020). The interface contacts were assigned between the femoral cartilage and menisci, between the menisci and medial and lateral tibial cartilages, and between the tibial and femoral cartilages, as previously described in the literature (Anwar et al., 2020). The friction coefficient of 0.02 was assumed in this study according to a previous biomechanical study (Mow et al., 1993).

**TABLE 1:** Material properties used in this study for the knee joint with validation in literature.

Model specification	Young’s modulus (MPa)	Poisson’s ratio	References
Cortical bone	7,300	0.3	Kim et al. (2011), Qiu et al. (2011)
Cancellous bone	1,100	0.26	Kim et al. (2011), Qiu et al. (2011)
Subchondral bone	3,000	0.3	Choi et al. (1990)
Cartilage	12	0.45	Hopkins et al. (2010)
Menisci	80	0.3	Hopkins et al. (2010)
Medial collateral ligament (MCL)	332	0.45	Innocenti et al. (2016)
Lateral collateral ligament (LCL)	345	0.45	Innocenti et al. (2016)
Anterior cruciate ligament (ACL)	169	0.45	Innocenti et al. (2016)
Posterior cruciate ligament (PCL)	177	0.45	Innocenti et al. (2016)

The foot bones were assigned an elastic modulus and Poisson’s ratio of 7,300 MPa and 0.3, respectively (Nakamura et al., 2016), and cartilages were assumed to have an elastic modulus and Poisson’s ratio of 45 MPa and 0.42, respectively (Dakin et al., 2000). The material properties of other ankle joint soft tissues are provided in Table 2. The effect of gravity was considered negligible in this study. The ankle cartilages were modeled using tie interaction with bones, as well as ligaments and bones. A normal interface was used between cartilage-to-cartilage interactions.

**TABLE 2:** Properties of ankle joint materials used in this study.

Model specification	Young’s modulus (MPa)	Poisson’s ratio	References
Foot bones	7,300	0.3	Crowninshield and Nakamura (1981)
Cartilage	45	0.42	Dakin et al. (2000)
TiCL	512	0.4	Siegler et al. (1988), Li et al. (2020)
ATiTL	184.5	0.4	Siegler et al. (1988), Li et al. (2020)
PTiTL	99.5	0.4	Siegler et al. (1988), Li et al. (2020)
ATFL	255.5	0.4	Siegler et al. (1988), Li et al. (2020)
PTFL	216.5	0.4	Siegler et al. (1988), Li et al. (2020)
AITiFL	260	0.4	Siegler et al. (1988), Li et al. (2020)
PITiFL	260	0.4	Siegler et al. (1988), Li et al. (2020)

TiCL, tibiocalcaneal ligament; ATiTL, anterior tibiotalar ligament; PTiTL, posterior tibiotalar ligament; ATFL, anterior talofibular ligament; PTFL, posterior talofibular ligament; AITiFL, anterior inferior tibiofibular ligament; and PITiFL, posterior inferior tibiofibular ligament.

The boundary conditions were established: a simulated force of 700 N was applied at the femoral head corresponding to the body weight of the individual to simulate the single-leg-standing condition (Mansur et al., 2022), whereas the distal ends of the foot were fixed in all degrees of freedom.

## Statistics

Statistical analysis was done using SPSS 16.0 (SPSS Inc., Chicago, IL). Means and standard deviations in ROI (region of interest) were determined using descriptive statistics. One-way ANOVA and Fisher’s LSD multiple comparison tests were performed as the protected *post hoc* procedures to determine the mean difference (Anwar et al., 2017; Anwar et al., 2018; Anwar et al., 2020; Anwar et al., 2020; Mansur et al., 2022). Bonferroni-corrected pairwise comparisons were additionally performed as a sensitivity analysis (Supplementary Tables S1-S5). Comparisons that remained significant after correction were interpreted as statistically significant. A *p-*value < 0.05 was considered statistically significant.

## Results

A detailed analysis of this study is described below.

### Stress analysis of tibial and talar cartilages

**FIGURE 4:**
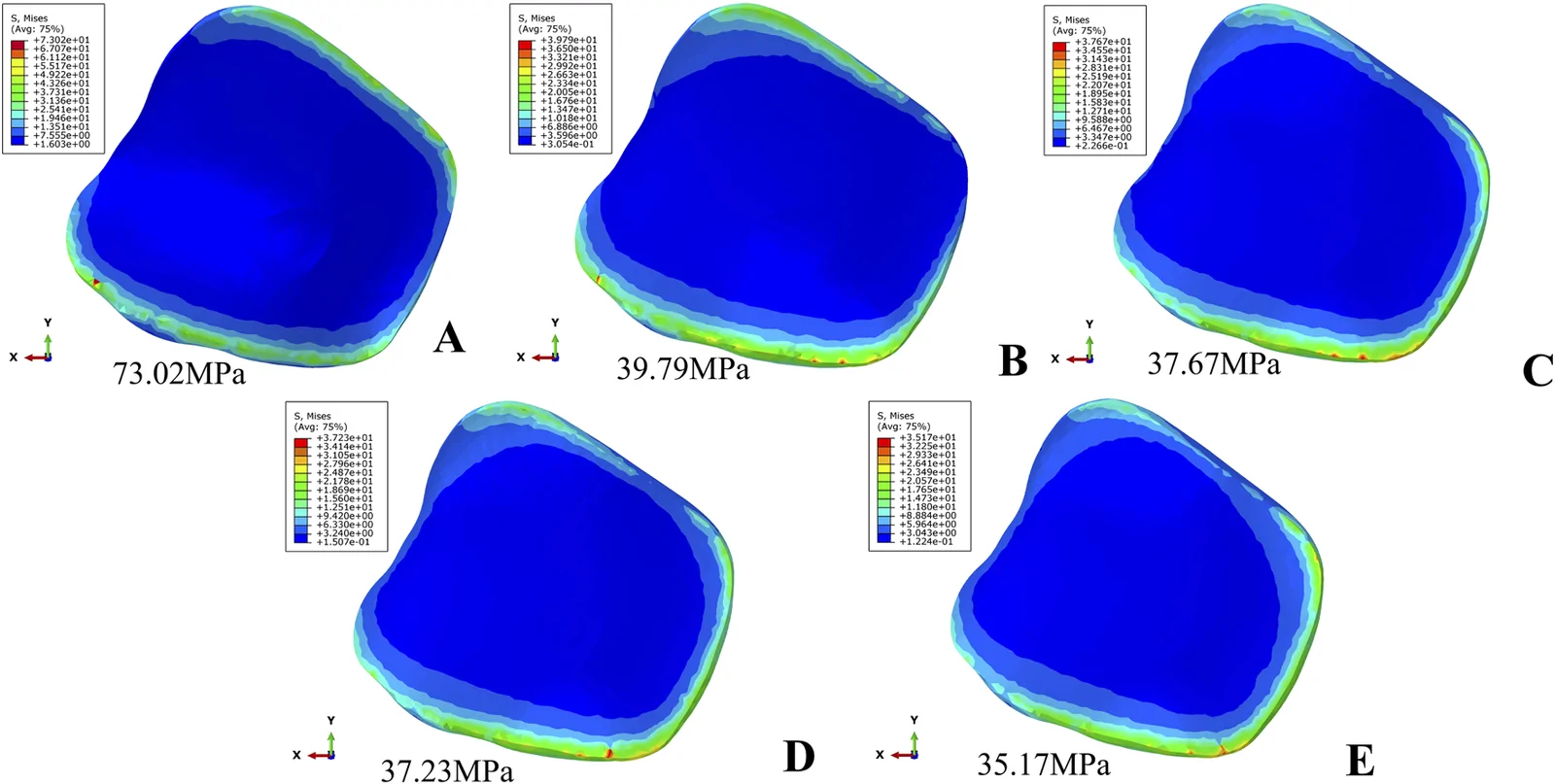
VMS analysis of tibial plafond cartilage in different models. **(A)** varus knee, **(B)** osteotomy model in neutral axis, **(C)** 3.5°, **(D)** 5.5° and **(E)** 7.5° over-corrected valgus models.

The peak stress of 73.02 MPa was noted in the tibial plafond cartilage of model A. It was progressively reduced to 39.79 MPa, 37.67 MPa, 37.23 MPa, and 35.17 MPa in models B, C, D, and E, respectively (*p* < 0.001), as depicted in Figure 4. The over-corrected models had different peak von Mises stress (VMS) values, but these were statistically insignificant (*p* > 0.05), as shown in Table 3.

**TABLE 3:** Tibial plafond cartilage VMS.

Model	​	Mean ± standard deviation (M ± SD)	Mean difference*	*p*-value
A	OA varus knee model	71.69 ± 1.21	35.52 (A–B) 37.14 (A–C) 37.55 (A–D)	0.000 (A–B) 0.000 (A–C) 0.000 (A–D)
B	Normal axis model	36.17 ± 3.80	1.61 (B–C) 2.03 (B–D) 3.92 (B–E)	0.519 (B–C) 0.421 (B–D) 0.136 (B–E)
C	3.5° valgus angle	34.55 ± 3.12	0.41 (C–D) 2.30 (C–E)	0.867 (C–D) 0.362 (C–E)
D	5.5° valgus angle	34.14 ± 3.09	1.89 (D–E)	0.452 (D–E)
E	7.5° valgus angle	32.25 ± 2.92	−39.44 (E–A)	0.000 (E–A)

* The mean difference is significant at the 0.05 level.

**FIGURE 5:**
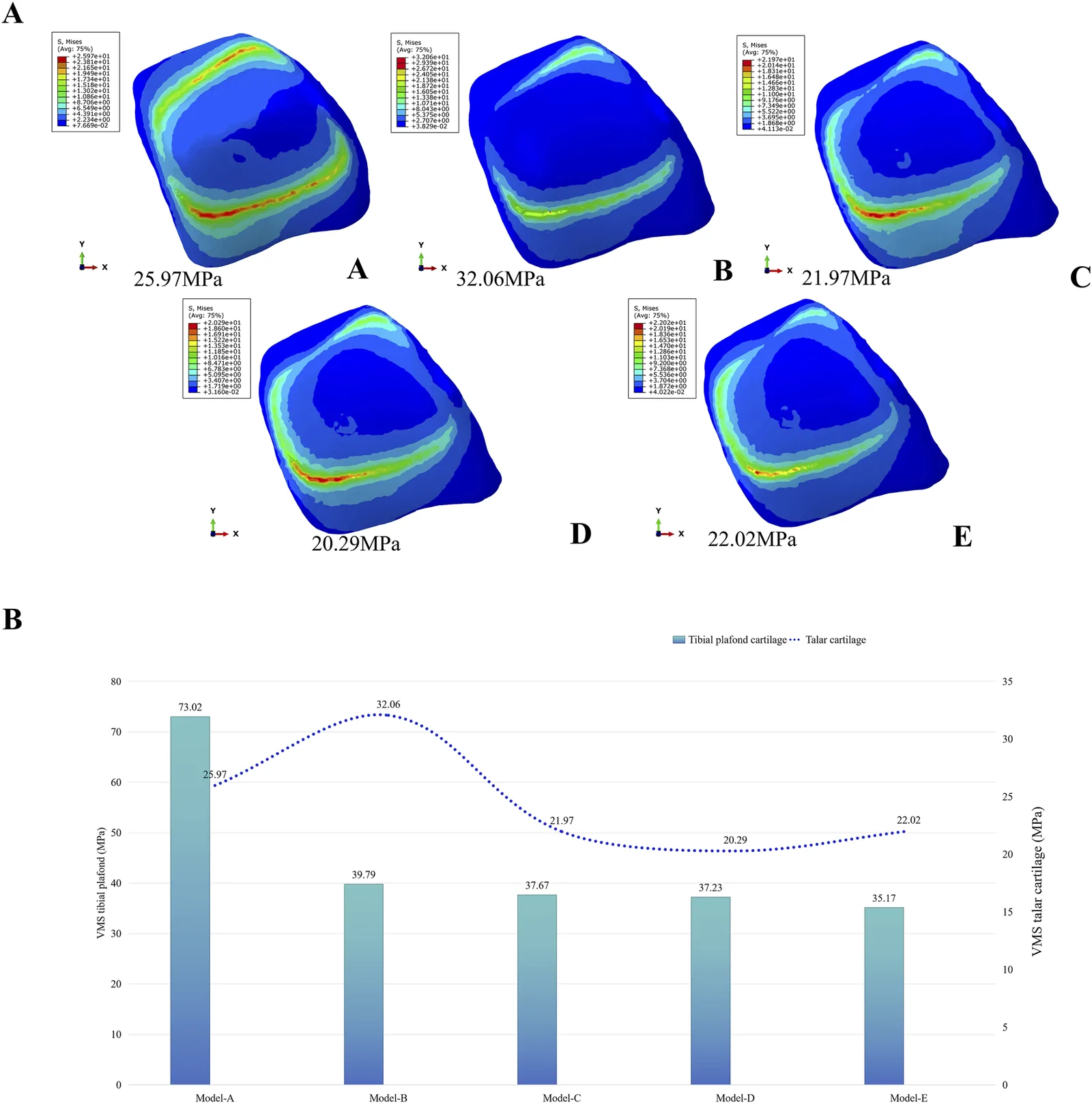
**(A)** VMS analysis of talar cartilage in different models. **(B)** Graphical comparison between tibial and talar components of ankle cartilages.

In talar cartilages, the VMS were relatively lower in model C (21.97 MPa), model D (20.29 MPa), and model E (22.02 MPa), moderate in model A (25.97 MPa), and highest in model B (32.06 MPa). Although the peak values in valgus correction groups were different, they were statistically insignificant (*p* > 0.05), as shown in Figure 5 and Table 4 for mean stress values and *p-*values.

**TABLE 4:** Talar cartilage VMS.

Model	​	Mean ± standard deviation (M ± SD)	Mean difference*	*p*-value
A	OA varus knee model	23.81 ± 2.16	−5.58 (A–B) 4.34 (A–C) 5.21 (A–D)	0.009 (A–B) 0.032 (A–C) 0.013 (A–D)
B	Normal axis model	29.39 ± 2.67	9.92 (B–C) 10.79 (B–D) 9.20 (B–E)	0.000 (B–C) 0.000 (B–D) 0.000 (B–E)
C	3.5° valgus angle	19.74 ± 2.16	0.87 (C–D) −0.72 (C–E)	0.626 (C–D) 0.689 (C–E)
D	5.5° valgus angle	18.60 ± 1.69	−1.59 (D–E)	0.382 (D–E)
E	7.5° valgus angle	20.19 ± 1.83	−3.62 (E–A)	0.064 (E–A)

* The mean difference is significant at the 0.05 level.

The reduction in talar cartilage VMS was significant in valgus-corrected models C and D compared to the varus knee model (*p* < 0.05), whereas it was non-significant in model E (*p* > 0.05); see Table 4.

### Tibial cartilage shear stress variations

**FIGURE 6:**
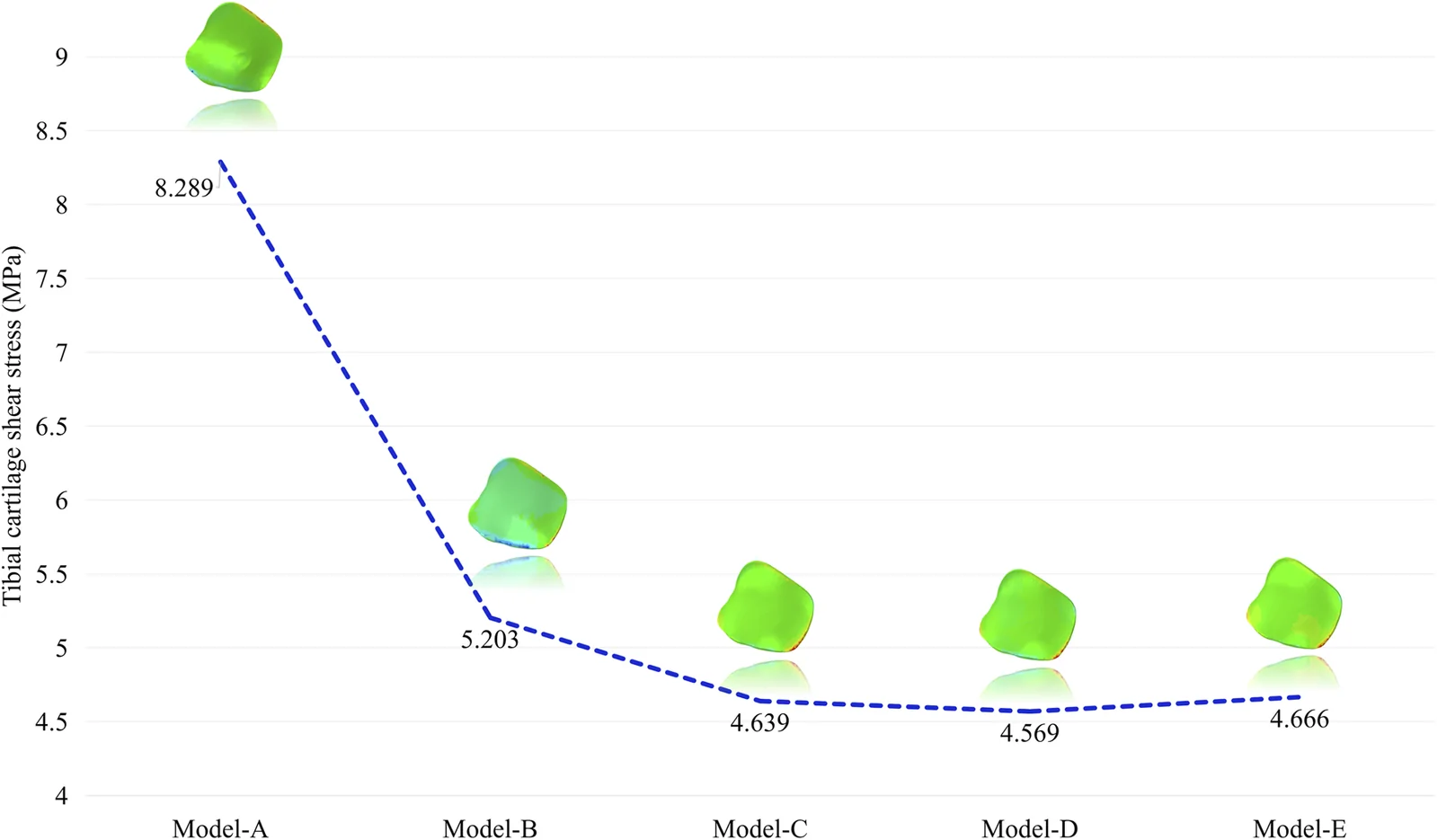
Shear stress analysis of tibial cartilage in various models.

Shear stress analysis of tibial cartilage showed that the peak shear stress of 8.289 MPa in model A was reduced to 5.203 MPa in the neutral axis model B (*p* < 0.05). It slightly decreased with small variations in the valgus models C, D, and E (*p* < 0.05), as shown in Figure 6 and Table 5.

**TABLE 5:** Tibial cartilage shear stress.

Model	​	Mean ± standard deviation (M ± SD)	Mean difference*	*p*-value
A	OA varus knee model	6.86 ± 1.43	2.37 (A–B) 3.07 (A–C) 3.06 (A–D)	0.014 (A–B) 0.003 (A–C) 0.003 (A–D)
B	Normal axis model	4.49 ± 0.72	0.70 (B–C) 0.69 (B–D) 0.74 (B–E)	0.397 (B–C) 0.408 (B–D) 0.374 (B–E)
C	3.5° valgus angle	3.78 ± 0.86	−0.02 (C–D) 0.04 (C–E)	0.983 (C–D) 0.965 (C–E)
D	5.5° valgus angle	3.80 ± 0.77	0.05 (D–E)	0.948 (D–E)
E	7.5° valgus angle	3.75 ± 0.92	−3.11 (E–A)	0.003 (E–A)

* The mean difference is significant at the 0.05 level.

### Subtalar cartilage pressure measurements

**FIGURE 7:**
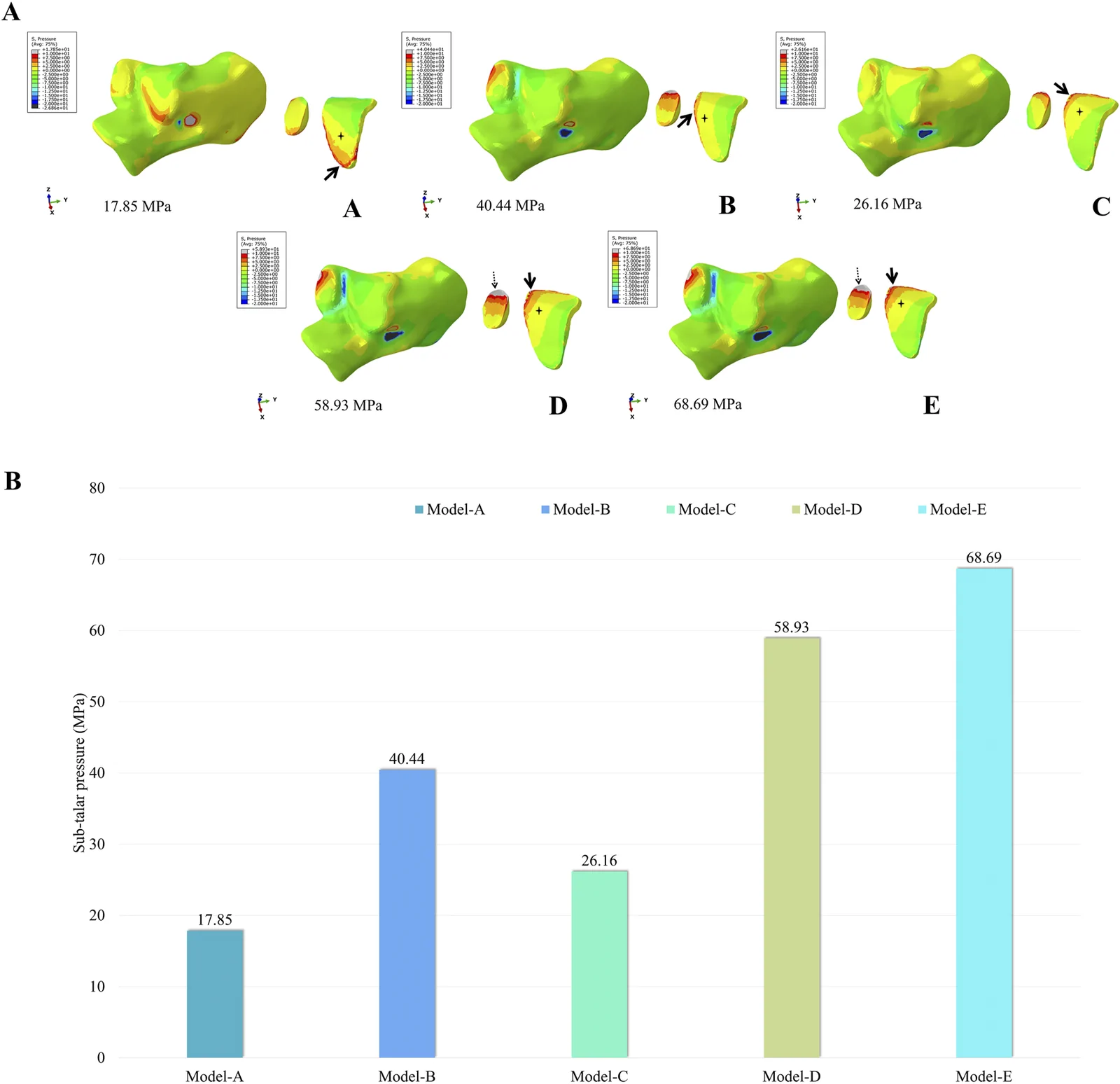
**(A)** Subtalar joint pressures in various models **(A–E)**. **(B)** Graphical representation of subtalar joint pressures.

Subtalar cartilage pressure analysis showed a minimum pressure of 17.85 MPa in the OA knee model. A lower pressure of 26.16 MPa was noted in the 3.5° valgus model. The neutral axis (model B) had 40.44 MPa (*p* < 0.05), and higher pressures of 58.93 MPa (*p* < 0.05) and 68.69 MPa (*p* < 0.05) were observed in model D and model E, respectively, as shown in Figures 7A,B and Table 6 for mean values.

**TABLE 6:** Statistical analysis of subtalar joint pressure.

Model	​	Mean ± standard deviation (M ± SD)	Mean difference*	*p*-value
A	OA varus knee model	10.59 ± 3.63	−24.37 (A–B) −15.40 (A–C) −28.03 (A–D)	0.011 (A–B) 0.097 (A–C) 0.004 (A–D)
B	Normal axis model	34.96 ± 6.53	8.97 (B–C) −3.66 (B–D) −10.60 (B–E)	0.32 (B–C) 0.69 (B–D) 0.25 (B–E)
C	3.5° valgus angle	25.99 ± 0.26	−12.64 (C–D) −19.58 (C–E)	0.169 (C–D) 0.038 (C–E)
D	5.5° valgus angle	38.63 ± 22.26	−6.94 (D–E)	0.444 (D–E)
E	7.5° valgus angle	45.57 ± 25.33	34.97 (E–A)	0.001 (E–A)

* The mean difference is significant at the 0.05 level.

### Tibial plafond stress patterns

**FIGURE 8:**
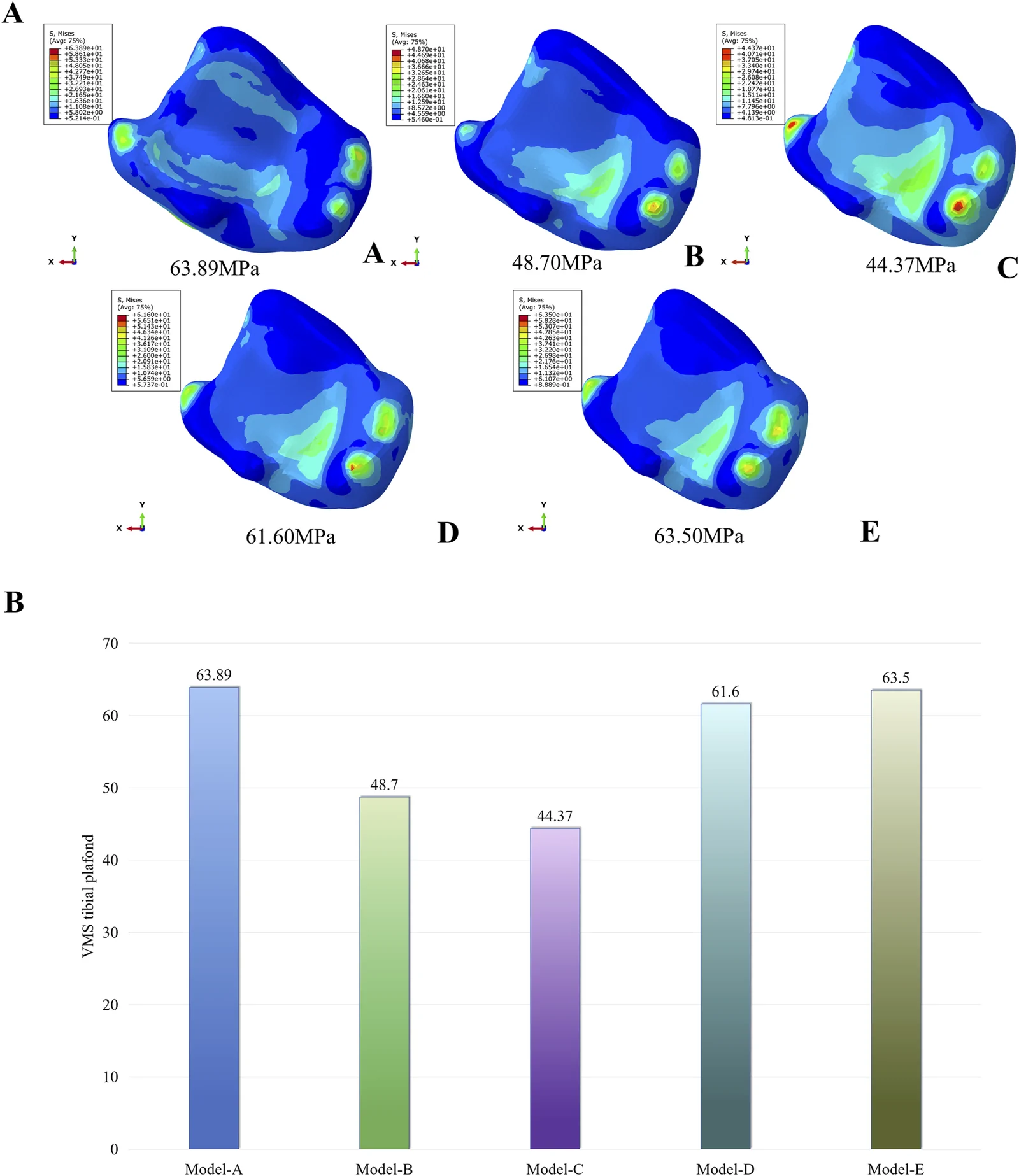
**(A)** Nephrogram showing tibial plafond bone VMS in different models. **(B)** Graphical representation.

Tibial plafond stress patterns are shown in Figure 8. The OA knee, model A, demonstrated the highest VMS of 63.89 MPa, whereas the lowest VMS of 44.37 MPa was observed in the 3.5° valgus model C (*p* < 0.001). The neutral axis model B had 48.70 MPa stress (*p* < 0.05), but the 5.5° and 7.5° valgus models D and E showed 61.60 MPa (*p* > 0.05) and 63.50 MPa (*p* = 0.492), respectively, as shown in Figure 8 and Table 7.

**TABLE 7:** Statistical analysis of tibial plafond VMS.

Group	​	Mean ± standard deviation (M ± SD)	Mean difference*	*p*-value
A	OA varus knee model	60.77 ± 3.00	16.08 (A–B) 20.06 (A–C) 4.26 (A–D)	0.001 (A–B) 0.000 (A–C) 0.251 (A–D)
B	Neutral axis model	44.69 ± 4.01	3.98 (B–C) −11.82 (B–D) −13.59 (B–E)	0.281 (B–C) 0.007 (B–D) 0.003 (B–E)
C	3.5° valgus angle	40.71 ± 3.66	−15.80 (C–D) −17.57 (C–E)	0.001 (C–D) 0.001 (C–E)
D	5.5° valgus angle	56.51 ± 5.09	−1.77 (D–E)	0.623 (D–E)
E	7.5° valgus angle	58.28 ± 5.22	−2.49 (E–A)	0.492 (E–A)

* The mean difference is significant at the 0.05 level.

## Discussion

The results of this FEA study showed the highest stress in the tibial plafond of the OA knee model A and a reduction in plafond stress in the neutral axis model B. This decrease in stress was further continued in the 3.5° valgus model C (44.37 MPa). After that, stress was increased with further increment in valgus angles, as shown in Figure 8. It means that the tibial plafond bone was under great stress in the varus knee model A (mean VMS: 60.77 MPa), and this stress was reduced in both the neutral axis model B (mean VMS: 44.69 MPa) and in the 3.5° valgus model C, with a mean VMS of 40.71 MPa (*p* < 0.001), as given in Table 7. We can also find that the medial area of the plafond was involved prominently in valgus knee models (see Figure 8D,E). This is because the foot and ankle are compensated in the varus position during the valgus knee deformity (Reilingh et al., 2010; Bae et al., 2020). Therefore, this varus ankle position caused accumulation of more stress on the medial side of the tibial plafond. In the neutral axis and 3.5° valgus models B and C, the foot adjusted in neutral and slight varus angulation. Therefore, the stress was shifted from more lateral (in OA varus knee model A) toward the central and central medial portions of the tibial plafond (see Figures 8B,C).

Moreover, the stress changes in distal tibial cartilages were highest in model A (73.02 MPa) and reduced to 39.79 MPa in the neutral axis model B. However, this reduction further continued to 37.67 MPa, 37.23 MPa, and 35.17 MPa in the 3.5°, 5.5°, and 7.5° valgus models C, D, and E, respectively. Therefore, the highest mean stress of 71.69 MPa in the varus knee model A may cause damage to distal tibial cartilage in the ankle joint (Anwar et al., 2020). Though the stress was reduced further in model E, this reduction was not statistically significant between the 3.5°, 5.5°, and 7.5° valgus models (*p* > 0.05). Only significant changes were observed in the OA knee model A (*p* < 0.05) compared with mean stresses in other models, as shown in Figure 4 and Table 3. Suero et al. (2015) conducted a cadaveric experimental study and concluded that moderate to large changes in proximal tibial alignment result in significantly decreased tibiotalar contact surface area and a significant decrease in intraarticular ankle pressures. Similar trends of decreasing distal tibial cartilage stress were observed in our study, as shown in Figures 4C–E, with decreasing cartilage stress in progressive valgus models.

The mean talar cartilage VMS was significantly lower in model C (19.74 MPa) and model D (18.60 MPa) compared to the mean stress of 23.81 MPa in model A (*p* < 0.05), and significantly higher in model B (29.39 MPa) (*p* < 0.05). Although the mean stress in talar cartilage of model E was lower (20.19 MPa) than the OA model, it was non-significant (*p* > 0.05), as shown in Table 4.

Moreover, comparing the highest mean tibial shear stress in model A (6.86 MPa), models B, C, D, and E showed mean shear stresses of 4.49 MPa, 3.78 MPa, 3.80 MPa, and 3.75 MPa (*p* < 0.05), respectively, as shown in Table 5 and Figure 6. The implication is that this higher shear stress in the OA knee model’s ankle joint cartilage can initiate and speed the articular cartilage damage (Anwar et al., 2020).

Subtalar joint pressure analysis showed that although the pressure was lowest in the OA model (17.85 MPa), the subtalar cartilage pressure area was concentrated on the more lateral side, as represented by the arrow and star in Figures 7A,A. This finding is in accordance with the results of a previous experimental cadaveric study conducted by Krause et al. (2017). They also concluded that in varus knee deformities, the subtalar pressure was more lateralized. In the neutral axis model, we can see that mostly the central area on the subtalar cartilage showed the highest pressure (Figures 7A,B). Furthermore, the more medialization of subtalar pressure was noted in valgus models (represented by arrows in Figures 7A,C–E). These findings were also in accordance with a previous experimental study (Krause et al., 2017).

Even though the maximum values of pressure in models C to E were higher than those of model A, the affected area in model A (and also its location on the calcaneum) was greater than in other models (represented by a star in Figure 7A,A). Another finding was the shifting of stress position from the lateral subtalar joint to medial, as shown in Figures 7A,A–F. This might be due to the compensatory varus response in the ankle joint in over-corrected valgus HTO models.

Some previous clinical studies concluded that over-corrected knee osteotomy with 8° valgus led to ankle pain and mild osteoarthritis of the ankle joint (Elson et al., 2013; Jeong and Soohoo, 2014). After that, restoration of the normal angle of the ankle joint was achieved by a medial closing supra-malleolar osteotomy (Elson et al., 2013; Jeong and Soohoo, 2014). In Figure 7B, we can find that model E with 7.5° valgus showed the highest value of peak subtalar pressure among all the models. Therefore, concerning these previous clinical studies (Elson et al., 2013; Jeong and Soohoo, 2014), our findings also demonstrated similar trends of subtalar pressure and might be a possible explanation of ankle joint osteoarthritis. In a finite element analysis, Anwar et al. (2020) demonstrated that increased stress can cause a degenerative effect on cartilage. In previous cadaveric biomechanical studies, researchers posited that repetitive increased pressure loading could damage the joints and cause degenerative changes (McKinley et al., 2008; Tochigi et al., 2008; Hunt et al., 2015).

Similarly, the highest peak pressure in model E (as represented by bold and dotted arrows in Figures 7A,D,E) might cause sub-talar articular cartilage deterioration.

Similar to other finite element studies, the limitations of this study should be acknowledged. In this study, we conducted a simulation of an extended knee under an axial force. Therefore, the dynamic behavior of the models in gait-cycle loading was not studied. Second, the muscle forces were not included in this study. The model components were assumed to behave as homogeneous isotropic materials, as proposed by previous finite element studies (Anwar et al., 2020; Li et al., 2024). The cadaveric experimental studies also had these limitations due to a lack of *in vivo* muscular forces simulation (Suero et al., 2017) and the biostatic axial loading experimental conditions, while ignoring dynamic joint movements (Suero et al., 2015). Irrespective of these limitations, 3D modeling, meshing, material properties, and boundary conditions have been validated with the literature. Therefore, the present model is more appropriate for evaluating relative differences among correction angles. However, direct subject-specific validation against cadaveric experimental data or clinical studies was not available.

## Conclusion

The varus deformity of the knee and its consequent corrections using various mechanical axis angles in high tibial osteotomy affected the ankle joint. The shear component of stress was highest in the ankle cartilage of the varus knee model and was reduced in osteotomy models. Moreover, tibial plafond stress was higher both in varus and extreme valgus correction models. The varus knee deformity caused the lateralization of subtalar pressure, and valgus correction resulted in medialization of subtalar pressure. Clinically, these may lead to ankle pain in a varus-deformed knee joint, and higher valgus correction should be acknowledged. Mild valgus correction, particularly the 3.5° valgus model, showed relatively lower and more favorable ankle stress than the varus and excessive valgus models. The authors advocate further cadaveric or clinical studies to evaluate the results of this controlled parametric simulation study.

## Data Availability

The raw data supporting the conclusions of this article will be made available by the authors, without undue reservation.

## Supplementary material

SUPPLEMENTARY FIGURE S1
**(A)** Representation of different mesh sizes used in the convergence study. **(B)** Mesh sensitivity analysis curves for various parameters.
