# Supplementary material for: Effects of different HTO correction angles on ankle joint biomechanics in 3D varus knee model: a patient-specific simulation study
Source: Front Bioeng Biotechnol. 2026 Aug 25;14:1808741. doi: 10.3389/fbioe.2026.1808741 (PMC13550838; doi:10.3389/fbioe.2026.1808741)
Supplement: Supplementary file 1 [file Table1.docx]

**Supplementary Tables**

**Supplementary Table:1 Tibial plafond cartilage VMS**

| Models |  | Mean ± Standard Deviation (M±SD) | Mean Difference* | P-value | Bonferroni Correction  P-value |
| --- | --- | --- | --- | --- | --- |
| A | OA varus knee model | 71.69±1.21 | 35.52(A-B),  37.14(A-C),  37.55(A-D) | 0.000(A-B)  0.000(A-C)  0.000(A-D) | 0.000(A-B)  0.000(A-C)  0.000(A-D) |
| B | Normal axis model | 36.17±3.80 | 1.61(B-C)  2.03(B-D)  3.92(B-E) | 0.519(B-C)  0.421(B-D)  0.136(B-E) | 1.000(B-C)  1.000(B-D)  1.000(B-E) |
| C | 3.5°valgus angle | 34.55±3.12 | 0.41(C-D)  2.30(C-E) | 0.867(C-D)  0.362(C-E) | 1.000(C-D)  1.000(C-E) |
| D | 5.5° valgus angle | 34.14±3.09 | 1.89(D-E) | 0.452(D-E) | 1.000(D-E) |
| E | 7.5° valgus angle | 32.25±2.92 | -39.44(E-A) | 0.000(E-A) | 0.000(E-A) |

Note: * The mean difference is significant at the 0.05 level.

**Supplementary Table:2 Talar cartilage VMS**

| Models |  | Mean ± Standard Deviation (M±SD) | Mean Difference* | P-value | Bonferroni Correction  P-value |
| --- | --- | --- | --- | --- | --- |
| A | OA varus knee model | 23.81±2.16 | -5.58(A-B),  4.34(A-C),  5.21(A-D) | 0.009(A-B)  0.032(A-C)  0.013(A-D) | 0.024(A-B)  0.035(A-C)  0.015(A-D) |
| B | Normal axis model | 29.39±2.67 | 9.92(B-C)  10.79(B-D)  9.20(B-E) | 0.000(B-C)  0.000(B-D)  0.000(B-E) | 0.000(B-C)  0.000(B-D)  0.000(B-E) |
| C | 3.5°valgus angle | 19.74±2.16 | 0.87(C-D)  -0.72(C-E) | 0.626(C-D)  0.689(C-E) | 1.000(C-D)  1.000(C-E) |
| D | 5.5° valgus angle | 18.60±1.69 | -1.59(D-E) | 0.382(D-E) | 1.000(D-E) |
| E | 7.5° valgus angle | 20.19±1.83 | -3.62(E-A) | 0.064(E-A) | 0.086(E-A) |

Note: * The mean difference is significant at the 0.05 level.

**Supplementary Table:3 Tibial cartilage shear stress**

| Models |  | Mean ± Standard Deviation (M±SD) | Mean Difference* | P-value | Bonferroni Correction  P-value |
| --- | --- | --- | --- | --- | --- |
| A | OA varus knee model | 6.86±1.43 | 2.37(A-B),  3.07(A-C),  3.06(A-D) | 0.014(A-B)  0.003(A-C)  0.003(A-D) | 0.044(A-B)  0.009(A-C)  0.010(A-D) |
| B | Normal axis model | 4.49±0.72 | 0.70(B-C)  0.69(B-D)  0.74(B-E) | 0.397(B-C)  0.408(B-D)  0.374(B-E) | 1.000(B-C)  1.000(B-D)  1.000(B-E)) |
| C | 3.5°valgus angle | 3.78±0.86 | -0.02(C-D)  0.04(C-E) | 0.983(C-D)  0.965(C-E) | 1.000(C-D)  1.000(C-E) |
| D | 5.5° valgus angle | 3.80±0.77 | 0.05(D-E) | 0.948(D-E) | 1.000(D-E) |
| E | 7.5° valgus angle | 3.75±0.92 | -3.11(E-A) | 0.003(E-A) | 0.009(E-A) |

Note: * The mean difference is significant at the 0.05 level.

**Supplementary Table:4 Statistical analysis of subtalar joint pressure**

| Models |  | Mean ± Standard Deviation (M±SD) | Mean Difference* | P-value | Bonferroni Correction  P-value |
| --- | --- | --- | --- | --- | --- |
| A | OA varus knee model | 10.59±3.63 | -24.37(A-B),  -15.40(A-C),  -28.03(A-D), | 0.011(A-B), 0.097(A-C), 0.004(A-D) | 0.038(A-B),  0.814(A-C),  0.028(A-D) |
| B | Normal axis model | 34.96±6.53 | 8.97(B-C),  -3.66(B-D),  -10.60(B-E) | 0.32(B-C), 0.69(B-D), 0.25(B-E) | 1.000(B-C),  1.000(B-D),  0.733(B-E) |
| C | 3.5°valgus angle | 25.99±0.26 | -12.64(C-D),  -19.58(C-E) | 0.169(C-D), 0.038(C-E) | 1.000(C-D),  0.034(C-E) |
| D | 5.5° valgus angle | 38.63±22.26 | -6.94(D-E) | 0.444(D-E) | 0.931(D-E) |
| E | 7.5° valgus angle | 45.57±25.33 | 34.97(E-A) | 0.001(E-A) | 0.000(E-A) |

Note: * The mean difference is significant at the 0.05 level.

**Supplementary Table:5 Statistical analysis of tibial plafond VMS**

| Models |  | Mean ± Standard Deviation (M±SD) | Mean Difference* | P-value | Bonferroni Correction  P-value |
| --- | --- | --- | --- | --- | --- |
| A | OA varus knee model | 60.77±3.00 | 16.08(A-B),  20.06(A-C),  4.26(A-D) | 0.001(A-B)  0.000(A-C)  0.251(A-D) | 0.002(A-B)  0.001(A-C)  1.000(A-D) |
| B | Normal axis model | 44.69±4.01 | 3.98(B-C)  -11.82(B-D)  -13.59(B-E) | 0.281(B-C)  0.007(B-D)  0.003(B-E) | 1.000(B-C)  0.021(B-D)  0.008(B-E) |
| C | 3.5°valgus angle | 40.71±3.66 | -15.80(C-D)  -17.57(C-E) | 0.001(C-D)  0.001(C-E) | 0.008(C-D)  0.003(C-E) |
| D | 5.5° valgus angle | 56.51±5.09 | -1.77(D-E) | 0.623(D-E) | 1.000(D-E) |
| E | 7.5° valgus angle | 58.28±5.22 | -2.49(E-A) | 0.492(E-A) | 1.000(E-A) |

Note: * The mean difference is significant at the 0.05 level.
